# Regulation of Bacterial Manganese Homeostasis and Usage During Stress Responses and Pathogenesis

**DOI:** 10.3389/fmolb.2022.945724

**Published:** 2022-07-15

**Authors:** Julia E. Martin, Lauren S. Waters

**Affiliations:** ^1^ Department of Biological Sciences, Idaho State University, Pocatello, ID, United States; ^2^ Department of Chemistry, University of Wisconsin Oshkosh, Oshkosh, WI, United States

**Keywords:** manganese, metal homeostasis, regulation, bacterial pathogenesis, bacterial stress responses, zinc, metal transport, metal interplay

## Abstract

Manganese (Mn) plays a multifaceted role in the survival of pathogenic and symbiotic bacteria in eukaryotic hosts, and it is also important for free-living bacteria to grow in stressful environments. Previous research has uncovered components of the bacterial Mn homeostasis systems that control intracellular Mn levels, many of which are important for virulence. Multiple studies have also identified proteins that use Mn once it is inside the cell, including Mn-specific enzymes and enzymes transiently loaded with Mn for protection during oxidative stress. Emerging evidence continues to reveal proteins involved in maintaining Mn homeostasis, as well as enzymes that can bind Mn. For some of these enzymes, Mn serves as an essential cofactor. For other enzymes, mismetallation with Mn can lead to inactivation or poor activity. Some enzymes may even potentially be regulated by differential metallation with Mn or zinc (Zn). This review focuses on new developments in regulatory mechanisms that affect Mn homeostasis and usage, additional players in Mn import that increase bacterial survival during pathogenesis, and the interplay between Mn and other metals during Mn-responsive physiological processes. Lastly, we highlight lessons learned from fundamental research that are now being applied to bacterial interactions within larger microbial communities or eukaryotic hosts.

## Introduction

Like most metal micronutrients, Mn is both essential for viability and toxic in excess. Identification of bacterial Mn transporters and the capacity of eukaryotic hosts to manipulate bacterial Mn levels revealed that Mn regulation is important for bacterial virulence (Kehres and Maguire, 2003; Kehl-Fie and Skaar, 2010). Early studies established that bacteria actively acquire Mn to serve as a cofactor for diverse enzymes and provide protection against reactive oxygen species (ROS) (Kehres and Maguire, 2003). Proteins mediating Mn import were identified in the late 1990’s, followed by characterization of Mn-binding transcription factor families (DtxR and Fur variants) that regulated importer expression (Kehres and Maguire, 2003; Lisher and Giedroc, 2013; Chandrangsu et al., 2017). Thereafter, identification of Mn exporters led to characterization of a Mn-binding *cis-*acting RNA regulatory element (riboswitch) (Waters, 2020). Additional Mn-enzymes (Bosma et al., 2021) and regulatory mechanisms at the post-transcriptional and protein stability levels continue to be defined.

While the components of bacterial Mn homeostasis systems were being uncovered, mechanistic models of metal selectivity were developed to account for how proteins avoid mismetallation (Waldron and Robinson, 2009). In general, proteins are thought to acquire common transition metals (iron (Fe), Zn, and Mn) from distinct metal pools with differential exchangeability (e.g., a labile pool associated with small molecules such as phosphate vs. a less available pool sequestered in proteins) (Imlay, 2015; Foster et al., 2022). To prevent mismetallation, bacteria adjust intracellular metal pools so that proteins preferentially acquire their cognate metal.

**FIGURE 1:**
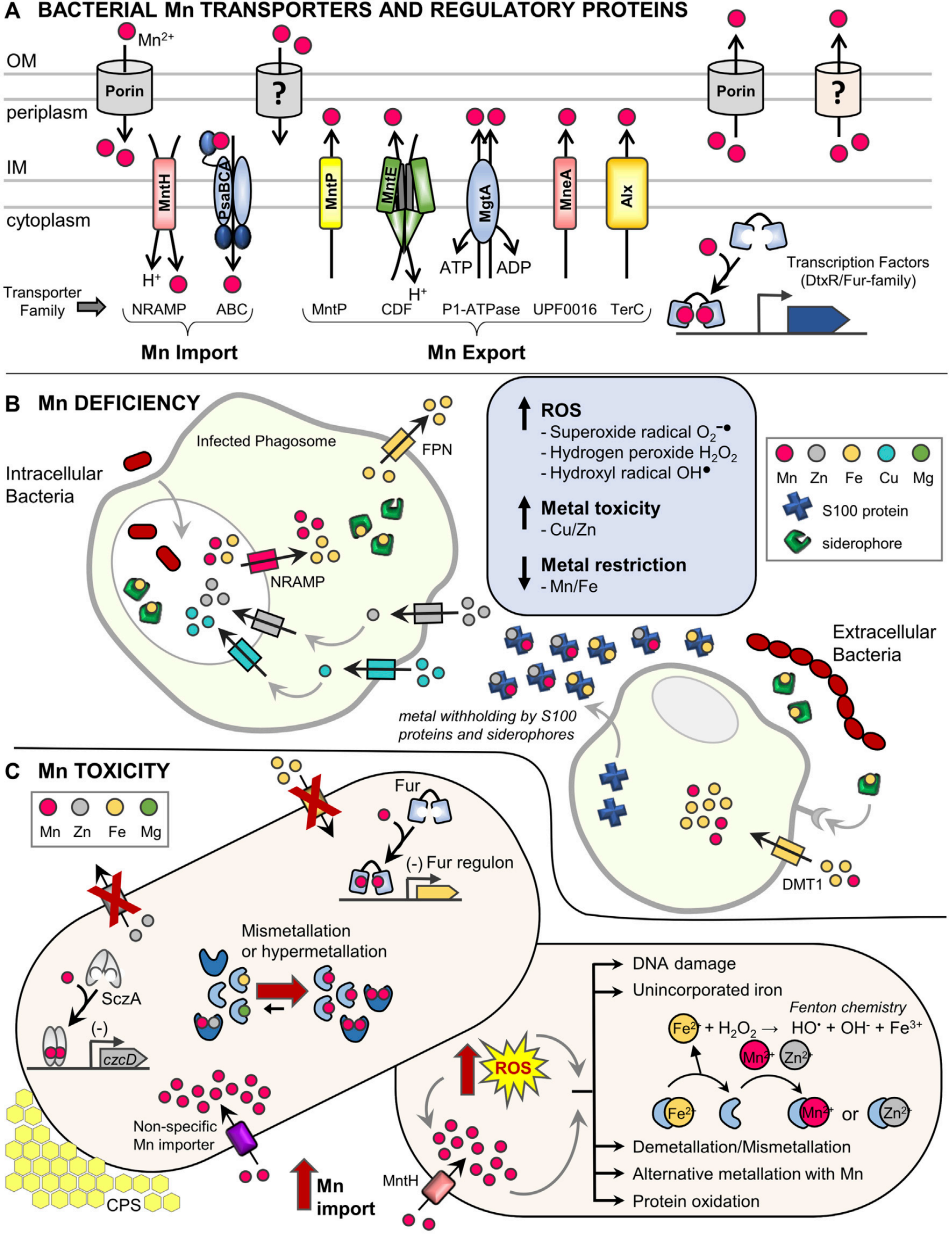
Dedicated manganese protein homeostasis machinery and impact of host defenses during bacterial pathogenesis. **(A)** Characterized bacterial Mn transporters and regulatory proteins. The bacterial Mn homeostasis protein machinery consists of dedicated, high-affinity Mn importers, exporters, and metal-binding transcription factors. Most bacteria contain at least one of either the NRAMP (MntH) or ABC transporter (PsaBCA) family importers in the inner membrane (IM), as well as one or more Mn-specific exporter types including the MntP, CDF, P1-type ATPase, UPF0016, and TerC families (Waters, 2020; Bosma et al., 2021). The distribution of Mn importers and exporters across bacterial species is complex and not fully understood (Zeinert et al., 2018). Expression of Mn transporters is commonly controlled by Mn-sensing transcription factors of the DtxR (MntR) or Fur (Mur) families (Capdevila et al., 2017). Post-transcriptional expression of several Mn exporters is also regulated by a Mn-sensing riboswitch, while others are constitutively expressed (Waters, 2020). Additional outer membrane (OM) components may also be involved in Mn transport (Hohle et al., 2011; Si et al., 2017; Jiang et al., 2020). Representative transporters are listed and transporter families are indicated below in brackets. NRAMP, natural resistance associated with macrophage protein; ABC, ATP-binding cassette; MntP, Mn transporter efflux pump; CDF, cation diffusion facilitator; P1-ATPase also known as E_1_-E_2_ ATPase; UPF0016, uncharacterized protein family 00016; TerC, tellurium resistant protein; DtxR, diphtheria toxin repressor; Fur, ferric uptake regulator. **(B)** Brief summary of metal homeostasis during a bacterial infection highlighting mechanisms that induce Mn deficiency. (*Left*) *Intracellular bacterial pathogens* residing inside of phagolysosomes of host phagocytes are subject to Mn and Fe deficiency when host NRAMP family transporters remove Mn and Fe from phagolysosome compartments. Additional host factors (siderophores that chelate Fe and the ferroportin (Fpn) exporter) further decrease available Fe levels (Chandrangsu et al., 2017; Antelo et al., 2021; Healy et al., 2021). Mn deficiency can be further exacerbated by intoxication with Zn and Cu by host transporters, which can lead to mismetallation of bacterial Mn- and Fe-using proteins causing cellular dysfunction (Djoko et al., 2015; Chandrangsu et al., 2017). *(Right) Extracellular bacterial pathogens* experience metal deficiency via sequestration of Mn and other metals by S100 proteins (e.g., calprotectin) and siderophores, as well as metal uptake into host phagocytic cells by DMT1 and other transporters (Healy et al., 2021). During Mn deficiency, bacteria suffer oxidative stress due to lack of Mn-dependent detoxification of ROS (Juttukonda and Skaar, 2015; Bosma et al., 2021). **(C)** Overview of Mn toxicity and oxidative stress in bacteria. *(Left)* Under conditions of *Mn excess*, Mn-bound MntR represses expression of Mn importers, while Mn-MntR and Mn-sensing riboswitches can induce expression of Mn exporters. Despite these efforts, high extracellular Mn may be inappropriately transported by other metal importers into cells (e.g., MgtE or ZIP transporters) (Grass et al., 2005; Hohle and O'Brian, 2014; Takeda et al., 2014). Elevated intracellular Mn can mismetallate proteins with diverse effects. Mn activation of transcription factors responsive to other metals (Fur, SczA) can lead to binding promoter DNA and cause inappropriate gene regulation (e.g., repression of Fe import systems and repression of Zn export), which further perturbs intracellular metal balances (Martin et al., 2015; Martin et al., 2017a). For some enzymes (Fe, Mg, or apoenzymes), mismetallation with Mn can lead to inactivation or poor activity (e.g., ferrochelatase, SodA, NrdAB (Cotruvo and Stubbe, 2012; Martin et al., 2015)). For other enzymes, binding with Mn can cause inappropriate activation or hyperactivation (e.g., PhpP, CpsB, Pgm (Martin et al., 2017b; McFarland et al., 2021)), which can lead to increased capsular polysaccharide (CPS) and cell elongation. (*Right*) During *oxidative stress*, Mn uptake is stimulated (Imlay, 2014). Unincorporated Fe^2+^ spontaneously reacts with H_2_O_2_ via Fenton chemistry, generating hydroxyl radicals that damage DNA and other macromolecules including iron-sulfur clusters, heme, and mononuclear Fe^2+^-containing proteins (Khademian and Imlay, 2021). Further metal homeostasis perturbation induces bacterial Mn acquisition and import resulting in mismetallation of proteins with Mn. Mismetallated proteins may have altered activity profiles ranging from inactivation to hyperactivation. In some cases, temporary alternative metallation with Mn during oxidative stress preserves limited enzyme activity of mononuclear Fe enzymes (Sobota and Imlay, 2011; Anjem and Imlay, 2012; Sobota et al., 2014). Additionally, low molecular weight Mn complexes likely contribute to ROS resistance (not shown) (Daly et al., 2010; McNaughton et al., 2010; Sharma et al., 2017).

Both Mn deficiency and toxicity are key problems that bacteria must avoid. How Mn deprivation occurs, particularly during pathogenesis, and its consequences for bacterial survival has been reviewed elsewhere (Juttukonda and Skaar, 2015; Sheldon and Skaar, 2019). In contrast, it is less clear how bacteria are subject to Mn toxicity during infection and what outcomes excess Mn has on physiology. Although the Mn-responsive regulatory and homeostasis machinery shared by most bacteria is well defined (Figure 1, and see (Bosma et al., 2021)), emerging regulatory mechanisms and additional specialized Mn importers relevant to pathogenesis continue to be uncovered.

Fundamental insights about bacterial physiology in pure culture have advanced understanding of the complex interplay in Mn usage and homeostasis in diverse ecosystems, and the field is ripe to examine the contribution of Mn-responsive systems during infection in the host. Here, we summarize recent advances in Mn stress responses and provide an outlook for the near future.

## New Regulatory Mechanisms Controlling Cellular Manganese Levels and Usage in Bacteria

**FIGURE 2:**
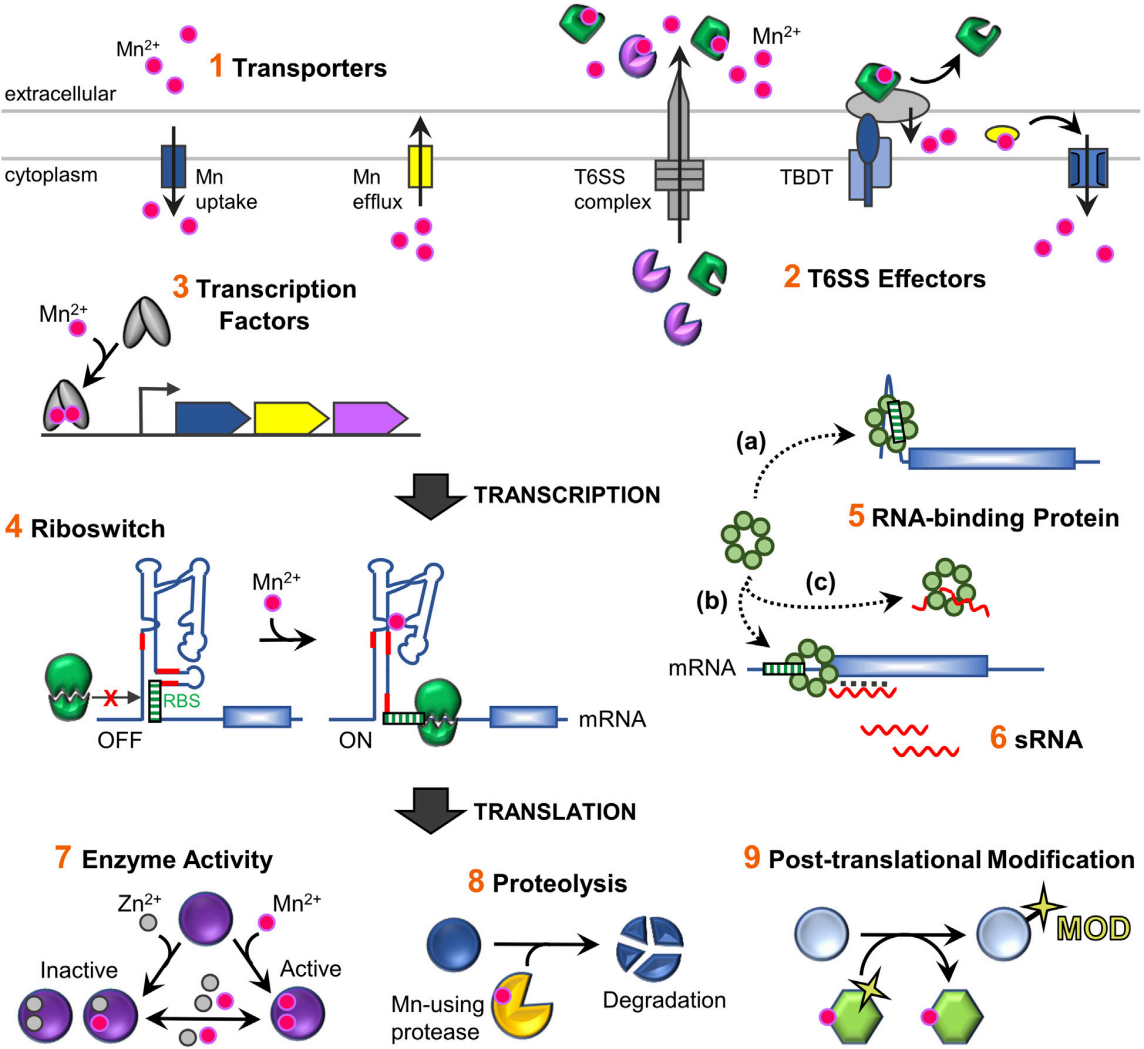
Schematic overview of the regulatory network for maintaining Mn homeostasis and its usage in bacterial cells. *Total intracellular levels* of Mn are controlled by 1) *regulated import and efflux* through dedicated Mn-specific transporters located in the inner membrane (refer to Figure 1A for representative transporter classes). In addition, 2) extracellular binding proteins (*T6SS effector proteins*) can capture extracellular Mn and deliver it to other uptake machinery (Yang et al., 2021). *Mn-sensing regulatory factors* control expression of Mn homeostasis genes, primarily encoding transporters, via conformational changes when bound to Mn. These include 3) protein transcription factors that *transcriptionally* regulate gene expression, as well as 4) RNA-based Mn-sensing riboswitch elements which provide *post-transcriptional* regulation *via* altering accessibility of the ribosome binding site (RBS) or an anti-termination event that allows transcription read-through of the coding region of the mRNA (not shown). Other *post-transcriptional* regulatory mechanism that affect mRNA stability and/or translation include 5) RNA-binding proteins (RBPs) such as CvfD (Sinha et al., 2020) and 6) sRNAs such as RsaC (Lalaouna et al., 2019). RBPs can act directly on mRNAs by binding to motifs present in the mRNA and/or enable sRNAs to base-pair with a mRNA. It is not currently known whether CvfD and RsaC require other factors to mediate their effects. In contrast to riboswitches, sRNAs do not typically directly sense environmental signals by binding ligands, but instead are expressed under specific environmental conditions (e.g., high or low Mn levels). Fluctuating intracellular Mn levels serve as an important role *post-translationally*, after nascent Mn-using proteins are synthesized. 7) The intracellular labile metal pool can influence the metallation status and *activity* of Mn-enzymes, such as PhpP, CspB, and Pgm (Martin et al., 2017b; McFarland et al., 2021). Such enzymes are active when bound to Mn, but become inactive with loss of metal or when replaced with Zn; reduced activity is observed with other metal cations. This process might also be aided by metal chaperone delivery factors during Mn deficient bacterial growth (not shown). 8) Metal-dependent protein factors, such as proteases, can directly control *protein levels*, as when the YqgP protease degrades the Mg transporter MgtA (Began et al., 2020). Other examples include Mn-binding transcription factors (PerR and Irr) that become oxidized when bound to Fe or heme, which leads to their degradation (not shown) (Lee and Helmann, 2006; Ahn and Baker, 2016; Nam et al., 2020). 9) Activated Mn-enzymes YdiU and PhpP may cause global physiological responses by modulating the activities of non-metal binding proteins *via post-translational protein modifications*, UMPylation and dephosphorylation, respectively (Martin et al., 2017b; Yang et al., 2020). Mn ions are designated as pink spheres; Zn as grey spheres.

Beyond established modes of regulation of Mn homeostasis (Figure 1), new findings are expanding the repertoire of regulatory approaches bacteria employ to maintain optimal cellular Mn levels (Figure 2). The fact that bacteria have evolved intricate measures to control Mn usage demonstrates its importance to cellular fitness.

### Manganese Regulation of Protein Synthesis

Mn homeostasis in bacteria is maintained by the expression and activities of Mn import and export machinery. Transcriptional regulation of Mn transporters has long been recognized to contribute to virulence (Ma et al., 2009). Beyond protein transcription factors, RNA-mediated elements can post-transcriptionally modulate gene expression. While several Mn-responsive riboswitches induce expression of downstream genes involved in Mn homeostasis (Dambach et al., 2015; Price et al., 2015; Martin et al., 2019; Waters, 2020), no other RNA-based regulation of Mn metabolism had been identified until recently, despite RNA-binding proteins (RBPs) and small RNAs (sRNAs) serving as important modulators of gene expression during pathogenesis (Oliva et al., 2015).

A newly characterized RNA-binding protein in *Streptococcus pneumoniae*, CvfD, contributes to virulence through several physiological pathways, including Mn and Zn homeostasis (Sinha et al., 2020). Deletion of *cvfD* caused increased transcription of the Mn-specific ABC-importer *psaBCA* and the Zn exporter *czcD*. Mn supplementation rescued the *cvfD* growth defect and restored capsule formation, which is dependent on the Mn:Zn ratio (McFarland et al., 2021), suggesting that CvfD helps maintain intracellular Mn:Zn balance. It is not yet known whether CvfD affects mRNA levels directly or by mediating sRNA base-pairing to target mRNAs.

The sRNA RsaC contributes to a Mn-sparing response in *Staphylococcus aureus.* RsaC is produced during Mn limitation from the 3’ end of the *mntABC* transcript encoding an ABC-type Mn importer (Lalaouna et al., 2019). Base-pairing of the RsaC sRNA with the ribosome-binding site of the *sodA* transcript reduces Mn-superoxide dismutase (SOD) protein levels. This shifts SOD activity away from Mn-dependence and towards reliance on the cambialistic Fe-using SOD, preserving bacterial resistance to ROS when Mn is scarce.

These examples set a precedent that *trans*-acting RNA regulation, well-established to regulate diverse stress responses (Oliva et al., 2015), is also used to control synthesis of proteins involved in Mn homeostasis and usage (Figure 2).

### Manganese Regulation of Protein Levels and Activity

Mn-responsive regulation of protein stability and covalent modifications can also impact Mn homeostasis and usage to promote bacterial survival. Recent examples include a Mn-activated protease and Mn-enzymes that add post-translational modifications to diverse non-metal-binding proteins. In *Bacillus subtilis*, elevated Mn can induce degradation of the magnesium (Mg) importer MgtE via activation of an intramembrane protease, YqgP (Began et al., 2020). Mn-mediated MgtE degradation reduces Mn stress, since Mn can also be adventitiously imported by MgtE during low Mg conditions. Cells may sacrifice MgtE to withstand Mn toxicity during periods of Mg starvation to avoid interference of high Mn with Mg-dependent processes and growth inhibition (Hohle and O'Brian, 2014; Pi et al., 2020). Although the Mn-sensing region of YqgP is not well conserved, these findings show that Mn-binding domains can be used to control activity of enzymes in a distinct mechanism from Mn serving as a cofactor. Moreover, intracellular Mn levels can be used to directly and rapidly control protein stability in response to metal fluctuations.

Mn-enzymes can also alter target protein activities via post-translational modifications. In *Salmonella typhimurium*, the stress-related pseudokinase YdiU modifies proteins via Mn-dependent UMP and AMP addition (Yang et al., 2020). Fur, MnSOD, and FeSOD were identified as targets of Mn-activated YdiU, as well as molecular chaperones and proteases. The UMPylation of chaperones prevented them from binding co-factors or client proteins, suggesting that YdiU may act as a global regulator that modulates protein activity (Yang et al., 2020). Similar Mn-dependent post-translational modifications involving phosphorylation cycling also affect enzyme activities in *S. pneumoniae* (Martin et al., 2017b).

Taken together, these findings suggest that Mn levels can affect physiology broadly and necessitates expanding our view of cellular processes that are responsive to environmental Mn changes beyond those using Mn-cofactored enzymes. Further studies may reveal how changing the balance of Mn with other metals alters the proteome.

## New Families of Manganese Importers Relevant During Pathogenesis

Although the suite of bacterial Mn transporters is well understood (Figure 1A), additional players continue to be identified. More types of Mn importers are emerging as important contributors to Mn uptake under specialized conditions, including stresses relevant to pathogenesis. These include ZIP family importers, outer membrane porins, and type VI secretion systems (T6SS).

Although primarily associated with Zn uptake, ZIP family importers can import additional metals, including Fe, Mn, and cobalt (Grass et al., 2005). Recently ZIP family members ZupT and TmpA were determined to be important under Mn stress during pathogenesis in *Salmonella* and *Streptococcus* (Yousuf et al., 2020; Puccio et al., 2022). *zupT* deletion in *Salmonella* eliminated residual Mn import in cells lacking the Mn-dedicated importers MntH and SitA and showed increased nitric oxide sensitivity (Yousuf et al., 2020). Similarly, deletion of *tmpA* in *Streptococcus* cells lacking the Mn importer SsaACB exacerbated a growth defect in serum and required higher exogenous Mn to rescue growth compared to the single Δ*ssaACB* mutant (Puccio et al., 2022). Together, these studies demonstrate an auxiliary role for ZIP family transporters in Mn uptake during virulence across diverse bacterial phyla.

Outer membrane proteins (OMPs) can also contribute to Mn transport. In *Bradyrhizobium,* the OMP MnoP directly mediates Mn import in proteoliposomes and is required *in vivo* for Mn uptake and survival during Mn scarcity (Hohle et al., 2011). In *Vibrio cholerae*, no dedicated Mn importers have been identified, but strains lacking the porin OmpU show decreased H_2_O_2_ resistance and reduced intracellular Mn (Jiang et al., 2020). Overexpression of *ompU* leads to increased intracellular Mn. Since *ompU* expression is induced by the ToxR virulence regulator, OmpU-mediated Mn import is likely important for *Vibrio* pathogenesis. These data suggest an underappreciated role for OMPs in Mn import and overall Mn homeostasis. A novel metal transport mechanism using T6SS effectors (TseM and TssS), together with an additional outer membrane TonB-dependent Mn transporter (MnoT), has also recently been described (Si et al., 2017; Yang et al., 2021; Zhu et al., 2021). In *Burkholderia,* TseM is secreted into the extracellular environment, where it specifically binds Mn and delivers it to MnoT for transport (Si et al., 2017). The small protein TssS produced by *Yersinia* is delivered into mammalian host cells, where it sequesters Mn. TssS-mediated Mn chelation reduces activity of the Mn-dependent enzyme cGAS, which activates the STING pathway of the innate immune response (Wang et al., 2018; Zhu et al., 2021). It is unclear if TssS also mediates Mn import into bacterial cells or if it primarily remains in the host cytoplasm in its Mn chelator role. Bacterial virulence and/or intracellular survival is reduced in both systems when the effectors or OM transporter are deleted (Si et al., 2017; Zhu et al., 2021). It will be interesting to see if additional Mn-binding proteins are found, as T6SS effector proteins are poorly characterized.

## Manganese Connection to Other Metals During Manganese-Responsive Physiological Processes

Mn influences dynamic and context-dependent processes such as bacterial virulence and biofilm formation. To do so, Mn acts in competition with other metals found within the intracellular metal milieu which is comprised of labile (freely accessible) metal ions and protein-bound (poorly accessible) metal complexes. The size and speciation of the labile metal pool is imprecisely known, especially for Mn which exists predominately in the spectroscopically hard-to-detect +2 oxidation state in cells and binds weakly to biomolecules (Irving and Williams, 1948). Nonetheless, the labile pool provides sufficient metal ions to ensure proper function of metalloenzymes, or—if perturbed—to cause mismetallation and Mn toxicity. We discuss here the interplay between Mn and other metal cations that serve as cofactors in bacterial proteins.

### Manganese and Zinc

Mn:Zn balance is an important factor that affects bacterial physiology and virulence (Chandrangsu et al., 2017; Bosma et al., 2021). Key Mn-enzymes in *Streptococcus* pathogenesis are strongly affected by perturbations in cellular Mn:Zn equilibrium (Martin et al., 2017b). Elevated Zn competitively inhibits Mn binding in enzyme active sites of PhpP and Pgm, resulting in inactivation. This disrupts cell division and capsule production, likely decreasing virulence. In contrast, higher Mn leads to enzyme hyperactivation and inappropriate function. These findings support that Mn-enzymes acquire metal by default (passive uptake from the labile metal pool), thus front-loading Mn homeostasis mechanisms as a key player in Mn metallation processes. This contrasts with many other characterized metalloenzymes that require chaperones or timely protein-folding pathways to specifically acquire their cognate metal (commonly Fe, Zn, and Cu); recent examples include enzyme-chaperone pairs CoxB-ScoI/PcuC and FolE-ZagA (Canonica et al., 2019; Chandrangsu et al., 2019).

Mn:Zn balance can also affect the virulence-related process of biofilm development. Recent evidence in *Klebsiella pneumoniae* indicates that elevated Zn caused by loss of the Zn exporter ZntA increases Mn levels and biofilm formation (Maunders et al., 2022). Elevated Mn also promotes biofilm formation by *B. subtilis* and *Lactobacillus plantarum* (Nozaka et al., 2014; Mhatre et al., 2016; Kovacs and Stanley-Wall, 2021). Suboptimal Mn concentrations increase biofilm aggregates of uropathogenic *E. coli* (UPEC) (Rowe et al., 2010). These biofilm aggregates can disperse as metal ions increase, leading to recurring infections. Although the mechanism by which Mn:Zn interplay induces bacterial biofilm-related processes remains undetermined, it is plausible that excess Mn might mismetallate Zn-metalloregulators (e.g., SzcA), causing cells to inappropriately perceive Zn limitation (Martin et al., 2017a).

### Manganese and Magnesium

Mg-dependent processes are known to be disrupted by excess Mn, likely by mismetallation of enzymes (Hohle and O'Brian, 2014). A recent genetic screen in *B. subtilis* identified targets of Mn intoxication in a highly Mn-sensitive strain (Pi et al., 2020). Suppressors conferring Mn tolerance included genes encoding a MerR-family transcriptional regulator (*yhdQ*), a putative Mg efflux transporter homologous to MpfA in *S. aureus* and CorC in *E. coli*, and the Mg-sensing riboswitch preceding the primary Mg importer MgtE. Loss of *mpfA* increased intracellular Mg levels, which might counteract inhibition by excess Mn, similar to what has been observed in *Saccharomyces cerevisiae* (Blackwell et al., 1998). It will be of significant interest to identify the Mg-dependent enzymes inhibited by excess Mn.

Like Mn:Zn, Mn:Mg can also influence bacterial attachment and biofilm maturation. Biofilm formation by *Pseudomonas aeruginosa* requires hydrolysis of (p)ppGpp by the RelA-SpoT family protein SAH, which exhibits strong cofactor preference for Mn over Mg *in vitro* (Steinchen et al., 2021). Several more RelA-SpoT family proteins show similar Mn preference (Ruwe et al., 2018). It remains to be determined if Mn has a direct role in biofilm production, is simply acting as a substitute for Mg, or is inappropriately binding to Mg-using proteins.

### Manganese and Iron

Fe-dependent processes and enzymes are also targets of mismetallation during Mn intoxication. Enzymes that use Fe as a Lewis-acid catalytic cofactor, such as SOD and ribonucleotide reductase, lose activity with Mn (Cotruvo and Stubbe, 2012); organisms may have Mn-dependent isoenzymes to compensate. Processes inactivated by Mn-mismetallation of Fe proteins include heme production (ferrochelatase) and Fe uptake (Fur) (Martin et al., 2015). However, Mn-mismetallation of other Fe-enzymes during oxidative stress when Fe is limited can protect enzymes from damage and be advantageous for growth (Imlay, 2014).

Historically, Mn:Fe balance has been studied in Fe-centric bacteria like *E. coli* where the intracellular Mn:Fe ratio is ≤ 0.1 and there is a larger number of Fe-using proteins (Bosma et al., 2021). *S. pneumoniae*, and possibly other Mn-centric bacteria with an intracellular Mn:Fe ratio ≥1, is also toxified by excess Mn, but via hyperactivation of Mn-enzymes (Martin et al., 2017b; McFarland et al., 2021). In *S. pneumoniae*, elevated Mn increases intracellular Fe levels during microaerophilic growth but significantly decreases Fe levels during anaerobiosis (Martin et al., 2017b). Since Mn-centric bacteria contain few Fe-using proteins, it is plausible that these Fe-enzymes have evolved metal-binding sites that preferentially select Fe over Mn or are preferentially expressed during anaerobiosis, thereby withstanding Mn toxicity.

### Manganese and Copper

Some evidence exists that excess Mn can interfere with copper (Cu) homeostasis and vice versa (Johnson et al., 2015; Martin et al., 2017b). In UPEC, Cu import was shown to confer Mn-dependent protection against ROS, while in *S. aureus* Cu import caused mismetallation of Mn-enzymes with Cu (Saenkham et al., 2020; Al-Tameemi et al., 2021). In UPEC, sublethal doses of Cu increased MnSOD levels which helped UPEC survive in macrophages (Saenkham et al., 2020). In the *S. aureus* study, which mimicked infection conditions in the mammalian host, a low Mn:high Cu ratio caused MntABC to inappropriately import sufficient Cu to cause toxicity, via mismetallation of NrdEF and the Fe-S dehydratase AcnA (Al-Tameemi et al., 2021).

Overall, high levels of a single metal can perturb the intracellular amounts of other metals (Figure 1C), causing complex physiological changes. The interplay between metals becomes particularly complex and significant as a bacterium navigates the dynamic process of infection inside a host.

## Lessons Learned From Experimental Biology Systems Applied to Pathogenesis

Fundamental studies of bacteria in pure culture have defined the strategies microbes use to acquire and remove Mn to combat Mn fluctuations, as well as the intracellular usage of Mn in enzymes and ROS detoxification. It is now commonly accepted that Mn availability, whether limited or in excess, can affect bacterial fitness. However, deciphering the functions of Mn is challenging as there is significant interplay between Mn and other metals. Many studies do not fully mimic the natural habitat where pathogenic bacteria derive Mn in hosts, but are essential for building fundamental understanding of how metals impact bacterial infection. With this knowledge, defined questions can be asked in the more complex multi-organism infection system.

In mammalian hosts, inflammation leads to rapid readjustment of the metal milieu to disrupt metal homeostasis, exploiting both essentiality and toxicity of metals to control bacterial infection. Host dietary metal uptake further influences the metal landscape (Lopez and Skaar, 2018). At the molecular level, single metal ion perturbations can significantly impact protein function. At the cellular level, metals are transported into and out of eukaryotic cells and their intracellular organelles to withhold metals or intoxicate bacteria (Figure 1B). Host siderophores and S100 proteins also sequester metal ions at the infection site. At the organismal level, host-mediated metal changes may manifest differently, depending on the site of infection and bacterial species involved (Lopez and Skaar, 2018). In a seminal study extending basic findings from pure culture into an animal model, mice fed a high-Mn diet had increased Mn levels that promoted *S. aureus* virulence in the heart by protecting bacteria from ROS, while not affecting other bacterial species in other niches (Juttukonda et al., 2017).

In addition to Mn deficiency imposed by nutritional immunity, it is clear that bacteria are vulnerable to excess Mn, given the ubiquity and numerous types of Mn exporters across bacteria. Pathogenic bacteria lacking Mn exporters also have reduced colonization and virulence (Johnsrude et al., 2019; Lam et al., 2020; Waters, 2020). However, it remains an open question where and how bacteria experience Mn toxicity within eukaryotic hosts. Although it is difficult to measure physiological levels of labile Mn in cells and tissues, monitoring Mn exporter expression levels during infection could provide some insight. More defined studies and new technologies are needed to elucidate Mn distribution under pathophysiological conditions. Such information will be instrumental in developing alternative antimicrobial therapies.

## Conclusions and Perspectives

In summary, new modes of regulation of Mn homeostasis and intracellular management demonstrate that Mn is critical for bacterial growth and virulence. We anticipate additional post-transcriptional and post-translational regulatory mechanisms affecting Mn levels and usage to be uncovered. We have likely only begun to understand the scope of how metal balances affect physiological responses through enzymes regulated by differential metallation. We look forward to pathogenesis studies which examine how Mn factors are used by bacteria growing in different host niches to counteract host immune defenses. New technologies that can quantify labile metal pools, as well as total Mn levels, in real-time in response to changing conditions would significantly aid the endeavor to characterize the bacteria-host interaction.

## References

[B1] Ahn B.-E. Baker T. A. (2016). Oxidization without Substrate Unfolding Triggers Proteolysis of the Peroxide-Sensor, PerR. Proc. Natl. Acad. Sci. U.S.A. 113 (1), E23–E31. 10.1073/pnas.1522687112 26677871 PMC4711837

[B2] Al‐Tameemi H. Beavers W. N. Norambuena J. Skaar E. P. Boyd J. M. (2021). *Staphylococcus aureus* Lacking a Functional MntABC Manganese Import System Has Increased Resistance to Copper. Mol. Microbiol. 115 (4), 554–573. 10.1111/mmi.14623 33034093 PMC8121185

[B3] Anjem A. Imlay J. A. (2012). Mononuclear Iron Enzymes Are Primary Targets of Hydrogen Peroxide Stress. J. Biol. Chem. 287 (19), 15544–15556. 10.1074/jbc.M111.330365 22411989 PMC3346116

[B4] Antelo G. T. Vila A. J. Giedroc D. P. Capdevila D. A. (2021). Molecular Evolution of Transition Metal Bioavailability at the Host-Pathogen Interface. Trends Microbiol. 29 (5), 441–457. 10.1016/j.tim.2020.08.001 32951986 PMC7969482

[B5] Began J. Cordier B. Březinová J. Delisle J. Hexnerová R. Srb P. (2020). Rhomboid Intramembrane Protease YqgP Licenses Bacterial Membrane Protein Quality Control as Adaptor of FtsH AAA Protease. EMBO J. 39 (10), e102935. 10.15252/embj.2019102935 31930742 PMC7231995

[B6] Blackwell K. J. Tobin J. M. Avery S. V. (1998). Manganese Toxicity towards *Saccharomyces cerevisiae* : Dependence on Intracellular and Extracellular Magnesium Concentrations. Appl. Microbiol. Biotechnol. 49 (6), 751–757. 10.1007/s002530051242 9684308

[B7] Bosma E. F. Rau M. H. van Gijtenbeek L. A. Siedler S. (2021). Regulation and Distinct Physiological Roles of Manganese in Bacteria. FEMS Microbiol. Rev. 45 (6), fuab028. 10.1093/femsre/fuab028 34037759 PMC8632737

[B8] Canonica F. Klose D. Ledermann R. Sauer M. M. Abicht H. K. Quade N. (2019). Structural Basis and Mechanism for Metallochaperone-Assisted Assembly of the Cu A Center in Cytochrome Oxidase. Sci. Adv. 5 (7), eaaw8478. 10.1126/sciadv.aaw8478 31392273 PMC6669012

[B9] Capdevila D. A. Edmonds K. A. Giedroc D. P. (2017). Metallochaperones and Metalloregulation in Bacteria. Essays Biochem. 61 (2), 177–200. 10.1042/EBC20160076 28487396 PMC5858914

[B10] Chandrangsu P. Huang X. Gaballa A. Helmann J. D. (2019). Bacillus subtilisFolE Is Sustained by the ZagA Zinc Metallochaperone and the Alarmone ZTP under Conditions of Zinc Deficiency. Mol. Microbiol. 112 (3), 751–765. 10.1111/mmi.14314 31132310 PMC6736736

[B11] Chandrangsu P. Rensing C. Helmann J. D. (2017). Metal Homeostasis and Resistance in Bacteria. Nat. Rev. Microbiol. 15 (6), 338–350. 10.1038/nrmicro.2017.15 28344348 PMC5963929

[B12] Cotruvo, Jr J. A. Jr. Stubbe J. (2012). Metallation and Mismetallation of Iron and Manganese Proteins *In Vitro* and *In Vivo*: the Class I Ribonucleotide Reductases as a Case Study. Metallomics 4 (10), 1020–1036. 10.1039/c2mt20142a 22991063 PMC3488304

[B13] Daly M. J. Gaidamakova E. K. Matrosova V. Y. Kiang J. G. Fukumoto R. Lee D.-Y. (2010). Small-molecule Antioxidant Proteome-Shields in *Deinococcus Radiodurans* . PLoS One 5 (9), e12570. 10.1371/journal.pone.0012570 20838443 PMC2933237

[B14] Dambach M. Sandoval M. Updegrove T. B. Anantharaman V. Aravind L. Waters L. S. (2015). The Ubiquitous yybP-ykoY Riboswitch Is a Manganese-Responsive Regulatory Element. Mol. Cell. 57 (6), 1099–1109. 10.1016/j.molcel.2015.01.035 25794618 PMC4376352

[B15] Djoko K. Y. Ong C.-l. Y. Walker M. J. McEwan A. G. (2015). The Role of Copper and Zinc Toxicity in Innate Immune Defense against Bacterial Pathogens. J. Biol. Chem. 290 (31), 18954–18961. 10.1074/jbc.R115.647099 26055706 PMC4521016

[B16] Foster A. W. Young T. R. Chivers P. T. Robinson N. J. (2022). Protein Metalation in Biology. Curr. Opin. Chem. Biol. 66, 102095. 10.1016/j.cbpa.2021.102095 34763208 PMC8867077

[B17] Grass G. Franke S. Taudte N. Nies D. H. Kucharski L. M. Maguire M. E. (2005). The Metal Permease ZupT from *Escherichia coli* Is a Transporter with a Broad Substrate Spectrum. J. Bacteriol. 187 (5), 1604–1611. 10.1128/jb.187.5.1604-1611.2005 15716430 PMC1064025

[B18] Healy C. Munoz-Wolf N. Strydom J. Faherty L. Williams N. C. Kenny S. (2021). Nutritional Immunity: the Impact of Metals on Lung Immune Cells and the Airway Microbiome during Chronic Respiratory Disease. Respir. Res. 22 (1), 133. 10.1186/s12931-021-01722-y 33926483 PMC8082489

[B19] Hohle T. H. Franck W. L. Stacey G. O'Brian M. R. (2011). Bacterial Outer Membrane Channel for Divalent Metal Ion Acquisition. Proc. Natl. Acad. Sci. U.S.A. 108 (37), 15390–15395. 10.1073/pnas.1110137108 21880957 PMC3174606

[B20] Hohle T. H. O'Brian M. R. (2014). Magnesium-dependent Processes Are Targets of Bacterial Manganese Toxicity. Mol. Microbiol. 93 (4), 736–747. 10.1111/mmi.12687 24975873 PMC4127137

[B21] Imlay J. A. (2015). “Common Mechanisms of Bacterial Metal Homeostasis,” in Trace Metals and Infectious Diseases. Editors Nriagu J. O. Skaar E. P. (Cambridge, MA: MIT Press). 10.7551/mitpress/9780262029193.003.0008 33886188

[B22] Imlay J. A. (2014). The Mismetallation of Enzymes during Oxidative Stress. J. Biol. Chem. 289 (41), 28121–28128. 10.1074/jbc.R114.588814 25160623 PMC4192467

[B71] Irving H. Williams R. J. P. (1948). Order of Stability of Metal Complexes. Nature 162, 746–747.

[B23] Jiang H.-h. Zhou Y. Liu M. Larios-Valencia J. Lee Z. Wang H. (2020). *Vibrio cholerae* Virulence Activator ToxR Regulates Manganese Transport and Resistance to Reactive Oxygen Species. Infect. Immun. 88 (3), e00944-19. 10.1128/IAI.00944-19 31871097 PMC7035944

[B24] Johnson M. D. L. Kehl-Fie T. E. Rosch J. W. (2015). Copper Intoxication Inhibits Aerobic Nucleotide Synthesis in Streptococcus Pneumoniae. Metallomics 7 (5), 786–794. 10.1039/c5mt00011d 25730343 PMC4431907

[B25] Johnsrude M. J. Pitzer J. E. Martin D. W. Roop R. M. 2nd (2019). The Cation Diffusion Facilitator Family Protein EmfA Confers Resistance to Manganese Toxicity in Brucella Abortus 2308 and Is an Essential Virulence Determinant in Mice. J. Bacteriol. 202 (1), e00357-19. 10.1128/JB.00357-19 31591273 PMC6932235

[B26] Juttukonda L. J. Berends E. T. M. Zackular J. P. Moore J. L. Stier M. T. Zhang Y. (2017). Dietary Manganese Promotes Staphylococcal Infection of the Heart. Cell. Host Microbe 22 (4), 531–542. e538. 10.1016/j.chom.2017.08.009 28943329 PMC5638708

[B27] Juttukonda L. J. Skaar E. P. (2015). Manganese Homeostasis and Utilization in Pathogenic Bacteria. Mol. Microbiol. 97 (2), 216–228. 10.1111/mmi.13034 25898914 PMC4631260

[B28] Kehl-Fie T. E. Skaar E. P. (2010). Nutritional Immunity beyond Iron: a Role for Manganese and Zinc. Curr. Opin. Chem. Biol. 14 (2), 218–224. 10.1016/j.cbpa.2009.11.008 20015678 PMC2847644

[B29] Kehres D. G. Maguire M. E. (2003). Emerging Themes in Manganese Transport, Biochemistry and Pathogenesis in Bacteria. FEMS Microbiol. Rev. 27 (2-3), 263–290. 10.1016/S0168-6445(03)00052-4 12829271

[B30] Khademian M. Imlay J. A. (2021). How Microbes Evolved to Tolerate Oxygen. Trends Microbiol. 29 (5), 428–440. 10.1016/j.tim.2020.10.001 33109411 PMC8043972

[B31] Kovács Á. T. Stanley-Wall N. R. (2021). Biofilm Dispersal for Spore Release in Bacillus Subtilis. J. Bacteriol. 203 (14), e0019221. 10.1128/JB.00192-21 33927051 PMC8223919

[B32] Lalaouna D. Baude J. Wu Z. Tomasini A. Chicher J. Marzi S. (2019). RsaC sRNA Modulates the Oxidative Stress Response of *Staphylococcus aureus* during Manganese Starvation. Nucleic Acids Res. 47 (18), 9871–9887. 10.1093/nar/gkz728 31504767 PMC6765141

[B33] Lam L. N. Wong J. J. Chong K. K. L. Kline K. A. (2020). *Enterococcus faecalis* Manganese Exporter MntE Alleviates Manganese Toxicity and Is Required for Mouse Gastrointestinal Colonization. Infect. Immun. 88 (6), e00058-20. 10.1128/IAI.00058-20 32229614 PMC7240088

[B34] Lee J.-W. Helmann J. D. (2006). The PerR Transcription Factor Senses H2O2 by Metal-Catalysed Histidine Oxidation. Nature 440 (7082), 363–367. 10.1038/nature04537 16541078

[B35] Lisher J. P. Giedroc D. P. (2013). Manganese Acquisition and Homeostasis at the Host-Pathogen Interface. Front. Cell. Infect. Microbiol. 3, 91. 10.3389/fcimb.2013.00091 24367765 PMC3851752

[B36] Lopez C. A. Skaar E. P. (2018). The Impact of Dietary Transition Metals on Host-Bacterial Interactions. Cell. Host Microbe 23 (6), 737–748. 10.1016/j.chom.2018.05.008 29902439 PMC6007885

[B37] Ma Z. Jacobsen F. E. Giedroc D. P. (2009). Coordination Chemistry of Bacterial Metal Transport and Sensing. Chem. Rev. 109 (10), 4644–4681. 10.1021/cr900077w 19788177 PMC2783614

[B38] Martin J. E. Edmonds K. A. Bruce K. E. Campanello G. C. Eijkelkamp B. A. Brazel E. B. (2017a). The Zinc Efflux Activator S Cz A Protects S Treptococcus Pneumoniae Serotype 2 D 39 from Intracellular Zinc Toxicity. Mol. Microbiol. 104 (4), 636–651. 10.1111/mmi.13654 28249108 PMC5426980

[B39] Martin J. E. Le M. T. Bhattarai N. Capdevila D. A. Shen J. Winkler M. E. (2019). A Mn-Sensing Riboswitch Activates Expression of a Mn2+/Ca2+ ATPase Transporter in Streptococcus. Nucleic Acids Res. 47 (13), 6885–6899. 10.1093/nar/gkz494 31165873 PMC6649816

[B40] Martin J. E. Lisher J. P. Winkler M. E. Giedroc D. P. (2017b). Perturbation of Manganese Metabolism Disrupts Cell Division inStreptococcus Pneumoniae. Mol. Microbiol. 104 (2), 334–348. 10.1111/mmi.13630 28127804 PMC5380469

[B41] Martin J. E. Waters L. S. Storz G. Imlay J. A. (2015). The *Escherichia coli* Small Protein MntS and Exporter MntP Optimize the Intracellular Concentration of Manganese. PLoS Genet. 11 (3), e1004977. 10.1371/journal.pgen.1004977 25774656 PMC4361602

[B42] Maunders E. A. Ganio K. Hayes A. J. Neville S. L. Davies M. R. Strugnell R. A. (2022). The Role of ZntA in *Klebsiella pneumoniae* Zinc Homeostasis. Microbiol. Spectr. 10 (1), e0177321. 10.1128/spectrum.01773-21 35019689 PMC8754117

[B43] McFarland A. L. Bhattarai N. Joseph M. Winkler M. E. Martin J. E. (2021). Cellular Mn/Zn Ratio Influences Phosphoglucomutase Activity and Capsule Production in Streptococcus Pneumoniae D39. J. Bacteriol. 203 (13), e0060220. 10.1128/JB.00602-20 33875543 PMC8316032

[B44] McNaughton R. L. Reddi A. R. Clement M. H. S. Sharma A. Barnese K. Rosenfeld L. (2010). Probing *In Vivo* Mn 2+ Speciation and Oxidative Stress Resistance in Yeast Cells with Electron-Nuclear Double Resonance Spectroscopy. Proc. Natl. Acad. Sci. U.S.A. 107 (35), 15335–15339. 10.1073/pnas.1009648107 20702768 PMC2932569

[B45] Mhatre E. Troszok A. Gallegos-Monterrosa R. Lindstädt S. Hölscher T. Kuipers O. P. (2016). The Impact of Manganese on Biofilm Development of Bacillus Subtilis. Microbiol. Read. 162 (8), 1468–1478. 10.1099/mic.0.000320 27267987

[B46] Nam D. Matsumoto Y. Uchida T. O'Brian M. R. Ishimori K. (2020). Mechanistic Insights into Heme-Mediated Transcriptional Regulation via a Bacterial Manganese-Binding Iron Regulator, Iron Response Regulator (Irr). J. Biol. Chem. 295 (32), 11316–11325. 10.1074/jbc.RA119.011855 32554810 PMC7415966

[B47] Nozaka S. Furukawa S. Sasaki M. Hirayama S. Ogihara H. Morinaga Y. (2014). Manganese Ion Increases LAB-Yeast Mixed-Species Biofilm Formation. Biosci. Microbiota Food Health 33 (2), 79–84. 10.12938/bmfh.33.79 25003021 PMC4081185

[B48] Oliva G. Sahr T. Buchrieser C. (2015). Small RNAs, 5′ UTR Elements and RNA-Binding Proteins in Intracellular Bacteria: Impact on Metabolism and Virulence. FEMS Microbiol. Rev. 39 (3), 331–349. 10.1093/femsre/fuv022 26009640

[B49] Pi H. Wendel B. M. Helmann J. D. (2020). Dysregulation of Magnesium Transport Protects Bacillus Subtilis against Manganese and Cobalt Intoxication. J. Bacteriol. 202 (7). 10.1128/JB.00711-19 PMC7167470 31964700

[B50] Price I. R. Gaballa A. Ding F. Helmann J. D. Ke A. (2015). Mn2+-Sensing Mechanisms of yybP-ykoY Orphan Riboswitches. Mol. Cell. 57 (6), 1110–1123. 10.1016/j.molcel.2015.02.016 25794619 PMC4703321

[B51] Puccio T. Kunka K. S. An S. S. Kitten T. (2022). Contribution of a ZIP‐family Protein to Manganese Uptake and Infective Endocarditis Virulence in Streptococcus Sanguinis. Mol. Microbiol. 117 (2), 353–374. 10.1111/mmi.14853 34855265 PMC8844249

[B52] Rowe M. C. Withers H. L. Swift S. (2010). Uropathogenic *Escherichia coli* Forms Biofilm Aggregates under Iron Restriction that Disperse upon the Supply of Iron. FEMS Microbiol. Lett. 307 (1), 102–109. 10.1111/j.1574-6968.2010.01968.x 20402788

[B53] Ruwe M. Rückert C. Kalinowski J. Persicke M. (2018). Functional Characterization of a Small Alarmone Hydrolase in Corynebacterium Glutamicum. Front. Microbiol. 9, 916. 10.3389/fmicb.2018.00916 29867827 PMC5954133

[B54] Saenkham P. Ritter M. Donati G. L. Subashchandrabose S. (2020). Copper Primes Adaptation of Uropathogenic *Escherichia coli* to Superoxide Stress by Activating Superoxide Dismutases. PLoS Pathog. 16 (8), e1008856. 10.1371/journal.ppat.1008856 32845936 PMC7478841

[B55] Sharma A. Gaidamakova E. K. Grichenko O. Matrosova V. Y. Hoeke V. Klimenkova P. (2017). Across the Tree of Life, Radiation Resistance Is Governed by Antioxidant Mn 2+ , Gauged by Paramagnetic Resonance. Proc. Natl. Acad. Sci. U.S.A. 114 (44), E9253–E9260. 10.1073/pnas.1713608114 29042516 PMC5676931

[B56] Sheldon J. R. Skaar E. P. (2019). Metals as Phagocyte Antimicrobial Effectors. Curr. Opin. Immunol. 60, 1–9. 10.1016/j.coi.2019.04.002 31063946 PMC6800623

[B57] Si M. Zhao C. Burkinshaw B. Zhang B. Wei D. Wang Y. (2017). Manganese Scavenging and Oxidative Stress Response Mediated by Type VI Secretion System in Burkholderia Thailandensis. Proc. Natl. Acad. Sci. U.S.A. 114 (11), E2233–E2242. 10.1073/pnas.1614902114 28242693 PMC5358365

[B58] Sinha D. Zheng J. J. Tsui H.-C. T. Richardson J. D. De Lay N. R. Winkler M. E. (2020). S1 Domain RNA-Binding Protein CvfD Is a New Posttranscriptional Regulator That Mediates Cold Sensitivity, Phosphate Transport, and Virulence in Streptococcus Pneumoniae D39. J. Bacteriol. 202 (18), e00245-20. 10.1128/JB.00245-20 32601068 PMC7925082

[B59] Sobota J. M. Gu M. Imlay J. A. (2014). Intracellular Hydrogen Peroxide and Superoxide Poison 3-Deoxy-D-Arabinoheptulosonate 7-phosphate Synthase, the First Committed Enzyme in the Aromatic Biosynthetic Pathway of *Escherichia coli* . J. Bacteriol. 196 (11), 1980–1991. 10.1128/JB.01573-14 24659765 PMC4010980

[B60] Sobota J. M. Imlay J. A. (2011). Iron Enzyme Ribulose-5-Phosphate 3-epimerase in *Escherichia coli* Is Rapidly Damaged by Hydrogen Peroxide but Can Be Protected by Manganese. Proc. Natl. Acad. Sci. U.S.A. 108 (13), 5402–5407. 10.1073/pnas.1100410108 21402925 PMC3069151

[B61] Steinchen W. Ahmad S. Valentini M. Eilers K. Majkini M. Altegoer F. (2021). Dual Role of a (p)ppGpp‐ and (p)ppApp‐degrading Enzyme in Biofilm Formation and Interbacterial Antagonism. Mol. Microbiol. 115 (6), 1339–1356. 10.1111/mmi.14684 33448498

[B62] Takeda H. Hattori M. Nishizawa T. Yamashita K. Shah S. T. A. Caffrey M. (2014). Structural Basis for Ion Selectivity Revealed by High-Resolution Crystal Structure of Mg2+ Channel MgtE. Nat. Commun. 5, 5374. 10.1038/ncomms6374 25367295 PMC4241985

[B63] Waldron K. J. Robinson N. J. (2009). How Do Bacterial Cells Ensure that Metalloproteins Get the Correct Metal? Nat. Rev. Microbiol. 7 (1), 25–35. 10.1038/nrmicro2057 19079350

[B64] Wang C. Guan Y. Lv M. Zhang R. Guo Z. Wei X. (2018). Manganese Increases the Sensitivity of the cGAS-STING Pathway for Double-Stranded DNA and Is Required for the Host Defense against DNA Viruses. Immunity 48 (4), 675–687. 10.1016/j.immuni.2018.03.017 29653696

[B65] Waters L. S. (2020). Bacterial Manganese Sensing and Homeostasis. Curr. Opin. Chem. Biol. 55, 96–102. 10.1016/j.cbpa.2020.01.003 32086169 PMC9997548

[B66] Yang X. Liu H. Zhang Y. Shen X. (2021). Roles of Type VI Secretion System in Transport of Metal Ions. Front. Microbiol. 12, 756136. 10.3389/fmicb.2021.756136 34803980 PMC8602904

[B67] Yang Y. Yue Y. Song N. Li C. Yuan Z. Wang Y. (2020). The YdiU Domain Modulates Bacterial Stress Signaling through Mn2+-Dependent UMPylation. Cell. Rep. 32 (12), 108161. 10.1016/j.celrep.2020.108161 32966796

[B68] Yousuf S. Karlinsey J. E. Neville S. L. McDevitt C. A. Libby S. J. Fang F. C. (2020). Manganese Import Protects *Salmonella enterica* Serovar Typhimurium against Nitrosative Stress. Metallomics 12 (11), 1791–1801. 10.1039/d0mt00178c 33078811 PMC7677218

[B69] Zeinert R. Martinez E. Schmitz J. Senn K. Usman B. Anantharaman V. (2018). Structure-function Analysis of Manganese Exporter Proteins across Bacteria. J. Biol. Chem. 293 (15), 5715–5730. 10.1074/jbc.M117.790717 29440394 PMC5900781

[B70] Zhu L. Xu L. Wang C. Li C. Li M. Liu Q. (2021). T6SS Translocates a Micropeptide to Suppress STING-Mediated Innate Immunity by Sequestering Manganese. Proc. Natl. Acad. Sci. U.S.A. 118 (42), e2103526118. 10.1073/pnas.2103526118 34625471 PMC8545469

